# Importance of the Functions of the Skin in the Pathology and Treatment of Tubercular Consumption

**Published:** 1860-08

**Authors:** 

**Affiliations:** St. Leonard’s


### IMPORTANCE OF THE FUNCTIONS OF THE SKIN

### IN THE PATHOLOGY AND TREATMENT OF TUBERCULAR CONSUMPTION.

BR DR. TOULMIN, OF ST. LEONARD'S.

The proximate cause of tubercle, is, in all cases, according to Dr. Toulmin, the breathing of impure air, and air in so small a quantity as to render it impure, especially during the night. The rich being subject to phthisis as well as the poor, he drew the attention of the Harveian Society to the importance of the respiratory functions of the skin, as proved by the fact that death resulted from closing the cutaneous pores artificially by varnishing the skin of rabbits and other animals, and suggested that from the coldness of the climate and other reasons, the better classes of society did not daily wash the whole surface of their bodies, and that the debris of the cuticle and the exuvia momentarily forming on the skin rendered the surface impervious to air. In this mode the same imperfect oxygenation of the blood ensues as is produced in the poorer classes by breathing mephitic air. For the removal of this state of skin, the only means of cure is free diaphoresis by artificial heat, which softens and expels large quantities of sebaceous matter, after the surface has been washed clean with soap and water.

Novel doctrines as to the nature and treatment of phthisis were enunciated; the treatment being considered under its hygienic and medical aspects; under the former, the patient was recommended to live in a high, dry, and marine atmosphere, on the downs rather than under them; and to be in the open air, devoting himself to suitable athletic exercises.

Medically, the treatment was mainly the use of the hot-air bath, the daily anointing of the whole surface with oleaginous matter, the keeping up open counter-irritation by setons or issues, and the use of tonics—each means of medication admirable in itself, but inefficacious if the hot-air bath, followed by aspersion of cold or tepid water should be omitted.—*Med. Times and Gazette, April*, 1860, *p.* 467.

